# GC-MS-Based Serum Metabolomic Investigations on the Ameliorative Effects of Polysaccharide from *Turpiniae folium* in Hyperlipidemia Rats

**DOI:** 10.1155/2021/9180635

**Published:** 2021-07-22

**Authors:** Xiao-lian Yang, Li Li, Tao-fu Zhang, Jing Deng, Xiu-lian Lin, Ya-mei Li, Bo-hou Xia, Li-mei Lin

**Affiliations:** ^1^Key Laboratory for Quality Evaluation of Bulk Herbs of Hunan Province, Hunan University of Chinese Medicine, Changsha 410208, China; ^2^School of Science, China Pharmaceutical University, Nanjing 211198, China

## Abstract

Hyperlipidemia, a typical metabolic disorder syndrome, can cause various cardiovascular diseases. The polysaccharides were found to have enormous potential in the therapy of hyperlipidemia. This study was aimed at evaluating the ameliorative effects of polysaccharide from *Turpiniae folium* (TFP) in rats with hyperlipidemia. A serum metabolomic method based on gas chromatography-mass spectrometry (GC-MS) was used to explore the detailed mechanism of TFP in rats with hyperlipidemia. The oxidative stress indicators, biochemical indexes, and inflammatory factors in serum and histopathological changes in the liver were also evaluated after 10-week oral administration of TFP in rats with high-fat diet-induced hyperlipidemia. TFP significantly relieved oxidative stress, inflammation, and liver histopathology and reduced blood lipid levels. Multivariate statistical approaches such as principal component analysis and orthogonal projection to latent structure square-discriminant analysis revealed clear separations of metabolic profiles among the control, HFD, and HFD+TFP groups, indicating a moderating effect of TFP on the metabolic disorders in rats with hyperlipidemia. Seven metabolites in serum, involved in glycine, serine, and threonine metabolism and aminoacyl-tRNA biosynthesis, were selected as potential biomarkers in rats with hyperlipidemia and regulated by TFP administration. It was concluded that TFP had remarkable potential for treating hyperlipidemia. These findings provided evidence for further understanding of the mechanism of action of TFP on hyperlipidemia.

## 1. Introduction

Hyperlipidemia is defined clinically as abnormally increased or decreased serum levels of total cholesterol (TC), triglyceride (TG), low-density lipoprotein cholesterol (LDL-c) levels, and high-density lipoprotein-cholesterol (HDL-c) *in vivo* [1]. The aberrant alteration in lipid levels is associated with various metabolic disorders related to diseases, such as essential hypertension, coronary heart disease, atherosclerosis, and diabetes [2, 3]. The correlative disease monitoring data reveal that, as the primary cause of death globally, cardiovascular and cerebrovascular diseases deriving from hyperlipidemia cause 4000 deaths per day [4]. Lipid-lowering drugs such as statins have made great achievements in treating hyperlipidemia. However, the undesirable side effects of statins reported recently make it imperative to excavate potential new drugs that can treat hyperlipidemia without any side effects [5]. Recently, natural polysaccharides have been proven to be a promising alternative to conventional therapy for hyperlipidemia [6].

Polysaccharides are polymeric carbohydrate structures containing long chains of monosaccharides with intermediate linkages. They have been applied for decades in various applications owing to their health benefits, biocompatibility, biodegradability, and relatively lower toxicity [7]. Moreover, they may be suitable for long-term supplementation. Comprehensive studies of polysaccharides are critical to the knowledge required for exploiting their potential. Polysaccharides are naturally found in plants, microbes (bacteria, fungi, and yeasts), seaweed, and animal sources [8]. Among these, plant polysaccharides contributed one-half of the amount [9]. Recently, polysaccharides from certain Chinese medicines have shown various biological activities, including hepatoprotective, antioxidant, anticancer, anti-inflammatory, and immunomodulation effects [10–13], and hence attracted increasing attention in medical and biochemical applications [14]. A growing body of evidence also showed that these polysaccharides possessed antihyperlipidemic, antioxidant, and hepatoprotective activities [15], as well as lipid-lowering effects [16].

*Turpiniae folium*, as a traditional Chinese medicine with a long history, is derived from *Turpinia arguta* Seem and often applied in treating tonsillitis and pharyngitis. It is characterized by high efficacy and low toxicity. Previous phytochemical and pharmacological investigations focused mainly on polar or moderately polar substances, such as flavonoids, alkaloids, and triterpenoids, and their anti-inflammatory, antibacterial, and immunomodulatory activities [17, 18]. Nevertheless, studies on highly polar, potentially active constituents of *T. folium*, such as polysaccharides, are limited. Moreover, previous studies established a comprehensive approach to extract *T. folium* polysaccharide (TFP) using the design of the experiment for the first time [19]. However, bioactivities or pharmacological effects of TFP are still unknown.

This study explored the possibility of the TFP-induced improvement in hyperlipidemia using the serum metabolomic approach based on GC-MS and elucidated underlying mechanisms. It was aimed at (1) evaluating the hepatoprotective effects of TFP against high-fat diet- (HFD-) induced liver injury using serum biochemical indicators and pathological observation, (2) performing metabolic profiles of TFP in hyperlipidemia, (3) screening potential biomarkers and analyzing the affected pathway and network, and (4) elucidating the mechanism underlying the hypolipidemic effect of TFP. This study was novel in investigating the effects of TFP on hyperlipidemia and the corresponding intervention mechanism.

## 2. Materials and Methods

### 2.1. Chemicals and Reagents

Enzyme-linked immunosorbent assay (ELISA) kits for examining biochemical indexes were purchased from Nanjing Jiancheng Bioengineering Institute (Jiangsu, China). Tumor necrosis factor *α* (TNF-*α*) was purchased from Lianke Biotechnology Co., Ltd. (Hangzhou, China). Lovastatin was obtained from Qilu Pharmaceutical Co., Ltd. (Jinan, China). Isoflurane was obtained from Ryward Life Technology Co., Ltd. (Changsha, China). Methoxyamine, *N*,*O*-bis(trimethylsilyl) trifluoroacetamide (BSTFA), 2-isopropylmalic acid, and pyridine were purchased from Sigma-Aldrich Co., Ltd. (Shanghai, China). Hematoxylin-eosin staining solution was provided by Wuhan Google Biotechnology (Wuhan, China). All high-fat diets were purchased from Slake Jingda Experimental Animal Co., Ltd. (Hunan, China). The commercial formulation of the basic diet was as follows: corn (20%), soybean meal (18%), wheat (38%), fish meal (10%), bran (5%), soybean oil (3%), maltodextrin (2%), and premix (4%). The commercial formulation of HFD was as follows: casein (25.84%), L-cystine (0.39%), corn (11.65%), maltodextrin (16.15%), sucrose (8.89%), cellulose (6.46%), soybean oil (3.28%), lard (20%), composite minerals (1.29%), calcium hydrogen phosphate (1.67%), calcium carbonate (0.71%), potassium citrate (2.13%), composite vitamin (1.29%), and hydrogen choline tartrate (0.25%).

### 2.2. Preparation of Polysaccharides of *T. folium*

*Turpiniae folium* was purchased from GaoQiao natural herbal special market (Changsha, China) and identified as dry leaves of *Turpinia arguta* Seem by Dr. Zhi Wang from the College of Pharmacy, Hunan University of Chinese Medicine (Changsha, China). TFP was prepared by a previously reported method [19]. The dried leaves of *T. folium* were crushed into powder (Disintegrator, HX-200A, Yongkang Hardware and Medical Instrument Plant, China) and sifted (80 meshes, Harbin Ouerfu Filter Material Co., Ltd., China). The as-prepared powder was subsequently extracted three times for 5 h every time with 80% ethanol at 60°C to remove small-molecule materials such as lipids, pigments, monosaccharides, and oligosaccharides. The insoluble parts were collected by filtration and dried at 60°C for 24 h.

The ultrasound-assisted enzymatic extraction of TFP was performed using an ultrasonic device (KQ-300DE, Kunshan Ultrasonic Instruments Co., Ltd., China). The defatted sample (500 g) was drawn out and put into a triangular flask for extraction. The specific liquid-to-solid ratio, enzyme concentration, pH value, ultrasonic power, extraction time, and temperature were set for the extraction. Thereafter, the solutions were centrifuged (5000 rpm for 10 min), and the supernatant was collected with no alcohol taste using a rotary evaporator at 50°C under vacuum. Then, dehydrated ethanol was added to a final concentration of 80% (*v*/*v*) (4°C for 24 h). After centrifugation (5000 rpm for 20 min), the precipitated polysaccharides were collected, washed with dehydrated ethanol, and lyophilized for crude polysaccharides (Lyophilizer, FD-1, Tokyo Physical and Chemical Machinery Co., Ltd.). Finally, the TFP content was 60.16% measured by the phenol-sulfuric acid method [20].

### 2.3. Animal Models and Experimental Design

**Figure 1:**
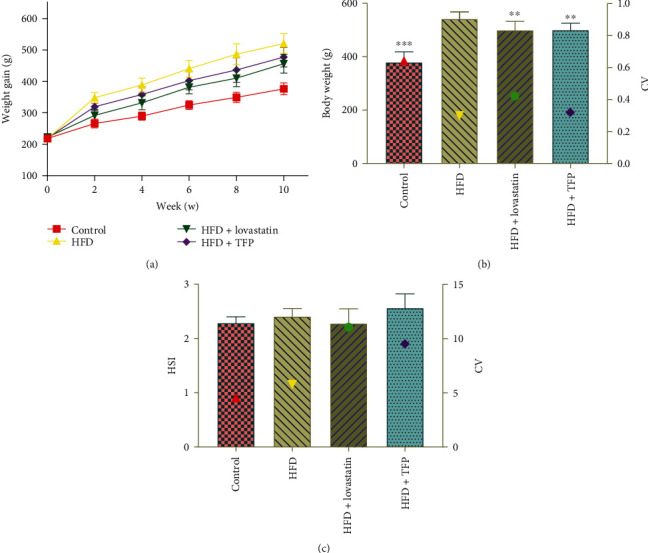
(a) Change of body weight of rats from four groups treated with different diets over time. (b) The body weight of rats from four groups: HFD+TFP group, HFD+lovastatin group, HFD group, and control group. (c) Hepatosomatic index (HSI) from four groups. The *y*-axis on the right represents the corresponding coefficient of variation (CV).

**Scheme 1:**
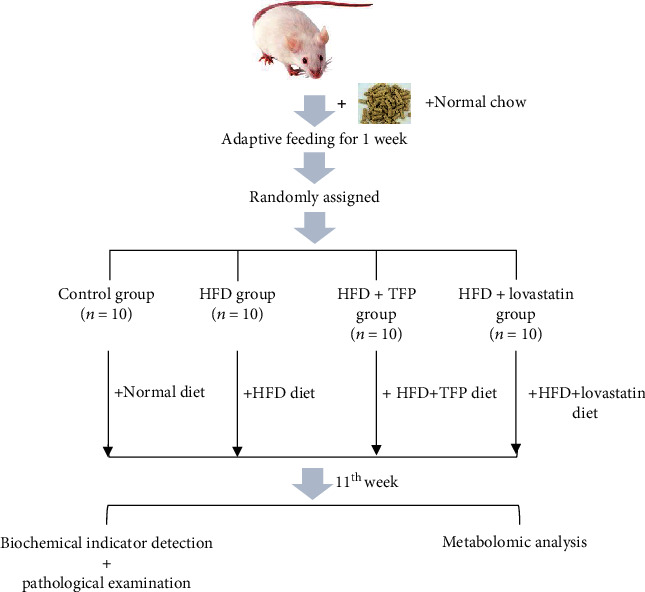
Schematic diagram of animal experiment design.

Forty male Sprague-Dawley rats (specific pathogen free (SPF) grade, 180 ± 20 g) were provided by Slake Jingda Laboratory Animal Co., Ltd. (Hunan, China) and housed in an environmentally controlled room at a constant temperature of 24 ± 1°C with a relative humidity of 50% ± 10% under a 12 h light-dark cycle. A normal chow diet and tap water were provided randomly. After acclimation for 1 week, the rats were randomly divided into groups based on the body weight; in the meantime, no significant difference was found in the body weight among the groups (the 0th week, Figure 1(a) shows that no significant difference in the body weight among groups was observed after the repeated measurement analysis of variance). Subsequently, the rats were randomly divided into four groups (*n* = 10 per group) as follows: normal control group (control group) supplied with a basal chow diet; the composition of the normal diet is presented in the section of the commercial formulation of basic diet in Chemicals and Reagents. The model control group (HFD group) was supplied with an HFD, and the corresponding composition of HFD diet is presented in the section of the commercial formulation of HFD in Chemicals and Reagents. The modeling method was based on a previously proposed method [21]. The positive control group (HFD+lovastatin group) was supplied with an HFD mixed with lovastatin (20 mg·kg^−1^); the treatment group (HFD+TFP group) was supplied with an HFD mixed with TFP (141 mg·kg^−1^). The dosage was administered according to the ratio table of equivalent doses converted from the reference human and rat body surface area. Specifically, the dosage of rats was calculated based on the formula: the dosage of rats = 15 g · 60 kg^−1^ · 9% · 6.25 = 141 mg · kg^−1^, where the 15 g represents the dosage of human clinically, while the 9% and 6.25 represent the yield rate of the polysaccharide extract from *T. folium* and the conversion coefficient of dosage of rats and human, respectively. In the paper, the aqueous solution of the polysaccharide extract from *T. folium* was used as the feeding of rats.  Scheme 1 shows the animal experimental design.

### 2.4. Sample Treatments

The body weight of all rats was monitored twice a week after continuous feeding for 10 weeks. All rats were anesthetized with isoflurane after 24 h of the last treatment. Blood samples of all rats were withdrawn from the abdominal aorta and further clotted for 1 h for a natural settlement. The supernatants after centrifugation (3000 rpm for 10 min) were collected in tubes and kept in a refrigerator at –80°C before the relative biochemical examination and metabolomic study. Then, all rats were sacrificed, and their livers were removed immediately, washed with physiological saline, weighed, and stored in a refrigerator at –80°C. The hepatosomatic index (HSI) was calculated based on the literature [22] (doi:10.1016/j.cbpb.2019.04.006) using the formula: HSI = [liver weight (g) · body weight (g)^−1^] · 100.

### 2.5. Serum Biochemical Assays

The activities of the marker enzymes, such as alanine aminotransferase (ALT), glutamic oxaloacetic transaminase (AST), and alkaline phosphatase (ALP), in serum samples, were measured using a multifunctional enzyme labeling instrument (Multiskan MK3, Thermo, Shanghai, China). TC, TG, LDL-c, and HDL-c were measured using corresponding protocols with a commercial automatic biochemical analyzer (SH-SH-04, Hitachi, Japan). Concurrently, the changes in blood glucose levels were measured in different groups of rats.

### 2.6. Serum Oxidative Stress Indicator and Inflammatory Factor Analysis

The activities of hepatic malondialdehyde (MDA), glutathione (GSH), and superoxide dismutase (SOD) were assayed using commercially available diagnostic kits. TNF-*α* was assayed using TNF-*α* ELISA kits following the manufacturer's protocols.

### 2.7. Histopathological Study on the Liver

The partition of liver tissues was removed and fixed immediately in 4% paraformaldehyde. The tissue block of the fixed target site was trimmed and smoothed, washed with running water for about 12 h, and then embedded in paraffin. After cooling and solidification, the wax block was removed from the encapsulation frame and repaired. Sections measuring 4 *μ*m were cut on a pathological section machine (RM2016, Leica Instruments Co., Ltd., Shanghai, China) and stained with hematoxylin-eosin for evaluating morphological changes under an optical microscope (Nikon Eclipse CI, Nikon, Japan). Liver steatosis, vacuole level, and liver pathological changes were examined on 200x and 400x images taken using a microscope. The remaining liver was immediately frozen in liquid nitrogen and stored in a refrigerator at –80°C until further analysis.

### 2.8. GC-MS Analysis

#### 2.8.1. Serum Sample Pretreatment

The serum samples were thawed at 4°C for 1 h, and 50 *μ*L of 2-isopropylmalic acid as the internal standard (1 mg·mL^−1^) was added to 100 *μ*L of serum samples and mixed using a vortex mixer. Then, 450 *μ*L of methanol was added to precipitate the proteins. The samples were placed for 8 min and centrifuged at 13,000 rpm for 10 min. The supernatant was transferred to a vial and evaporated to dryness under a gentle stream of nitrogen gas. Further, a 50 *μ*L pyridine solution of methoxyamine (20 mg·mL^−1^) was added to the dry residue, mixed using the vortex, and incubated at 70°C for 1 h. Thereafter, 100 *μ*L of the BSTFA derivatization reagent was added to the aforementioned mixture, vortex-mixed for 30 s, and incubated for 1 h at 70°C. Finally, the samples were placed at room temperature for 2 h and centrifuged for 8 min at 13,000 rpm. Further, 100 *μ*L of the supernatant was transferred to a 250 *μ*L GC vial for the GC-MS assay. The quality control samples (QCs) were prepared by mixing an equal volume of experimental samples for methodological investigation including precision, repeatability, and stability. The processing of QC samples was the same as the preparation of the aforementioned samples. Six QC samples were included in the analytical batch for monitoring GC-MS.

#### 2.8.2. GC-MS Procedure

The derivatized samples were separated on a nonpolar DB-5MS quartz capillary column (30 m × 0.25 mm × 0.25 *μ*m; Shimadzu, Japan) of a GC-MS spectrometer (GC-MS-QP2010, Shimadzu, Japan) using the following parameters: sampling volume, 1 *μ*L; carrier gas, helium (99.999%); flow rate, 1.0 mL·min^−1^; and split ratio, 10 : 1. The initial oven temperature was 70°C maintained for 4 min and then increased to 110°C at the rate of 20°C·min^−1^, 190°C at the rate of 8°C·min^−1^, and 270°C at the rate of 8°C·min^−1^ and maintained for 5 min. The inlet temperature was 280°C. The temperature of the electron impact ionization source was 200°C, the solvent delay time was 6.5 min, and the scanning range of mass spectrometry was 35–550 *m*/*z*. During the formal analysis, the sampling sequence of all samples was randomly arranged to avoid the run order effect and systematic variation attributed to instrument-based analyses. One QC sample was analyzed after every five sample injections.

#### 2.8.3. Data Processing

One-way analysis of variance, followed by the least significant difference test, of data was performed using SPSS for Windows version 25.0 (SPSS Inc., IL, USA). The results were deemed to be statistically significant at *P* < 0.05. The biochemical indexes in serum were analyzed using GraphPad Prism 8.0 (CA, USA) and presented as mean ± standard deviation. All raw mass spectrometry data were normalized using Mass Spectrometry-Data Independent Analysis (MS-DIAL) software for deconvolution, denoising, smoothing, peak identification, peak alignment, and peak filtering. Data calibration was performed using the internal standard. The correlation peaks were analyzed and identified based on the pyrolysis law of compounds by mass spectrometry. The variables with more missing values were removed by applying an 80% rule. The metabolites with relative standard deviation (RSD) for relative peak areas < 30% in the QC samples were retained to ensure the repeatability of the experiment. After the aforementioned processing, a data matrix was obtained in which the rows represented the samples and the columns represented the abundance of peaks of metabolites (equal to concentration).

### 2.9. Multivariate Statistical Analysis

The data matrix was imported into the website MetaboAnalyst 5.0 (http://www.metaboanalyst.ca/) for multivariate statistical analysis after the data were Pareto scaled and logarithmically transformed. Principal component analysis (PCA) was used to overview intrinsic dissimilarity among groups. Orthogonal projection to latent structure discriminant analysis (OPLS-DA) was also used to screen out differential biomarkers between groups. The quality of the models was assessed using model parameters *Q*^2^ (denoting predictability of the model) and *R*^2^ (evaluating the goodness of fit of the model). Moreover, permutation tests (*n* = 100 times) and tenfold cross-validation models were performed to validate the reliability and prediction performance of models to guard against the overfitting of the OPLS-DA model. Developing biomarkers is one of the methods of current metabonomic research [23]. In this study, multiple approaches were applied to screening the potential biomarkers to ensure the accuracy of the outcome.

The *P* value (threshold = 0.05) and fold change (FC) (threshold = 1.5) were calculated to visualize the metabolite differences between groups. The area under the receiver operating characteristic (AUC-ROC) curve was calculated using SigmaPlot 14.0 software to evaluate the classification performance of models [23]; a model was regarded as excellent for classification when the AUC-ROC exceeded 0.9 [24]. Subsequently, the differential metabolites were screened based on the projection importance of characteristic variable (VIP) > 1.0, FC > 1.5, and ROC curve analysis (AUC‐ROC > 0.9). Finally, the Venn plot, a frequently used method with high accuracy, was constructed for confirming the shared potential biomarkers [25]. This method possessed comprehensiveness and accuracy for screening differential metabolites [26]. The intersectional differential screened biomarkers using the aforementioned methods were all selected as potential biomarkers for the subsequent pathway analysis.

Moreover, the potential biomarkers were imported on the website MetaboAnalyst 5.0 for enrichment and pathway analysis. The related pathways with impact > 0.10 were regarded as the most relevant potential target pathways associated with the intervention mechanism of TFP [27].

## 3. Results

### 3.1. Effects of TFP on Body Weight and HSI of Rats with Hyperlipidemia

**Figure 2:**
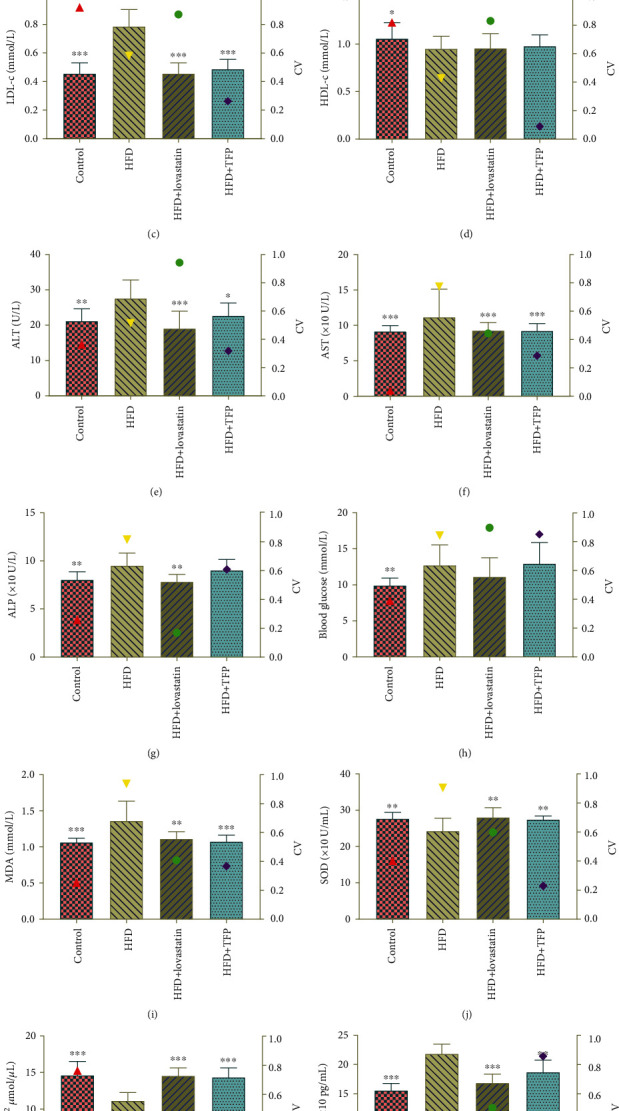
The serum lipid, AST, ALT, ALP, and blood glucose levels and the serum content of oxidative stress indicators of rats from four groups. The *y*-axis on the right represents the corresponding coefficient of variation (CV).

Weight gain is an intuitive parameter for HFD-induced hyperlipidemia. As shown in Figure 1, the body weight showed a significant decrease in both HFD+TFP and HFD+lovastatin groups compared with the HFD group, tending to be closed to that in the control group, after 10 weeks of HFD feeding (Figure 1(a)). In the 10th week, the body weight remarkably increased in the HFD group compared with the control group. This increase was significantly attenuated in the HFD+TFP group (*P* < 0.05). Intriguingly, all of the treatments showed no significant difference in HSI (liver weight (g) · body weight (g)^−1^ · 100) (Figure 2(c)), indicating a corresponding increase in the liver weight with the increase in the body weight.

### 3.2. Effects of TFP on the Serum Lipid Profile

The serum lipid profile is an important indicator for evaluating hyperlipidemia models. As shown in Figure 2, serum TC, TG, and LDL-c levels in the HFD group rose to higher levels with a significant difference, whereas the HDL-c level showed a slight reduction without statistical significance (*P* > 0.05) in the HFD group compared with the control group. This finding showed that the administration of an HFD excessively promoted serum TC, TG, and LDL-c levels, but the decrease in the serum HDL-c level was not susceptible to the HFD (*P* > 0.05), which was consistent with the reported findings [28]. Also, it was confirmed that the hyperlipidemia model was established successfully. Compared with the HFD group, the HFD+TFP group exhibited a significant reduction in the levels of serum TC, TG, and LDL-c, indicating that the lipid profile in rats with hyperlipidemia was significantly regulated after treatment with TFP. However, TFP showed no significant effect on the HDL-c level.

### 3.3. Effects of TFP on ALT, AST, ALP, and Blood Glucose Indicators in Serum

ALT is one of the most sensitive indicators of liver function. It is also used with AST as an indicator of hepatocyte damage and loss of functional integrity [29]. ALP mainly produced in the liver was often used as an adjunct marker for the diagnosis of hepatobiliary clinically. At the end of the experiment, the serum levels of ALT, AST, ALP, and blood glucose were analyzed using their respective protocols. As shown in Figure 2, ALT, AST, and ALP levels increased in the HFD group, whereas the ALT and AST levels significantly decreased in the HFD+TFP group. This finding implied that the administration of TFP remarkably attenuated liver injury. However, TFP had no significant effect on ALP, suggesting that ALP was not suitable as an indicator of liver function in this model [30]. Interestingly, the HFD significantly increased the blood glucose level (*P* < 0.01), but TFP intervention did not cause a decline in the blood glucose level with statistical significance in the HFD group.

### 3.4. Effect of TFP on Serum Oxidative Stress Indicator and Inflammatory Factors

MDA is usually used to evaluate tissue lipid peroxidation injury and the severity of a free radical attack [31]. SOD can eliminate free radicals from the body and prevent damage caused by lipid peroxidation [32]. GSH is a tripeptide composed of glutamic acid, glycine, and cysteine (Cys) and is important in the reduction reaction *in vivo*. The levels of MDA, SOD, and reduced GSH, together examined as the indicators of oxidative stress, as well as TNF-*α* levels, were assayed in this experiment. As shown in Figure 2(i), the MDA level significantly increased in the HFD group compared with the control group (*P* < 0.001). However, the increase in the HFD-induced MDA level could be ameliorated by TFP to the level in the control group, as manifested by the decline in the MDA level in the HFD+TFP group (*P* < 0.001). Contrary to the MDA levels, the SOD and GSH levels decreased in the HFD group compared with the control group (Figures 2(j) and 2(k)), demonstrating oxidant-antioxidant imbalance in rats with HFD-induced hyperlipidemia. The intervention of TFP significantly reversed the decreasing tendency of SOD and GSH levels and enhanced the antioxidant capacity, as evidenced by the increased SOD and GSH levels in the HFD+TFP group to the level in the control group. Similar to MDA results, as shown in Figure 2(l), the TNF-*α* level increased in the HFD group compared with the control group (*P* < 0.001). However, the HFD+TFP group showed a significant decline in the TNF-*α* level (*P* < 0.01) compared with the HFD group, indicating that TFP ameliorated the inflammatory reactions. Lovastatin used for treatment of rats also exhibited the protective effect of attenuating the increase in the HFD-induced TNF-*α* level.

### 3.5. Histological Investigations

**Figure 3:**
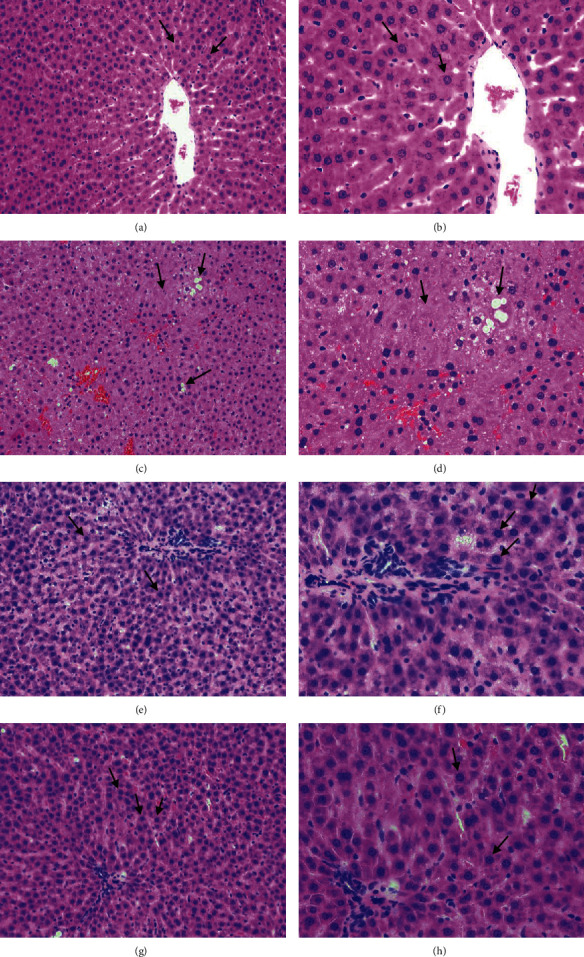
Histopathological section photographs of rat liver tissue slices for H&E analysis: (a, b) control group; (c, d) HFD group; (e, f) HFD+TFP group; (g, h) HFD+lovastatin group (magnification of H&E staining sections: the left column: 200x, the right column: 400x).

As shown in Figure 3, the closely packed hepatic lobules, cords, sinusoids, and hepatic cells with well-preserved cytoplasm, prominent nucleus, and no fatty degeneration were observed in the control group (Figures 3(a) and 3(b), where the arrow points). However, typical hyperlipidemia was observed in the HFD group, including extreme swelling of liver cells, disappeared cell nucleus, adipocytic degeneration, and lipid droplet accumulation (Figures 3(c) and 3(d), where the arrow points). However, the HFD+TFP group presented an amelioration on liver damage as manifested by the diminution of the fat vacuole area, attenuation of lipid droplet accumulation, and normalization of adipocytes (Figures 3(e) and 3(f), where the arrow points). The result was consistent with the findings of biochemical indicator measurements, further confirming the ameliorating effect of TFP on rats with hyperlipidemia.

### 3.6. Metabolomic Analysis

#### 3.6.1. Methodological Investigation and Preliminary Characterization of Metabolic Profiles

**Figure 4:**
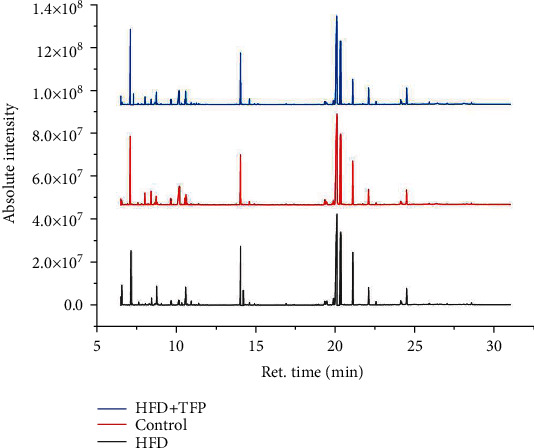
Total ion chromatogram of serum samples from the control group, HFD group, and HFD+TFP group. The lateral axis represents the retention time (ret. time).

**Figure 5:**
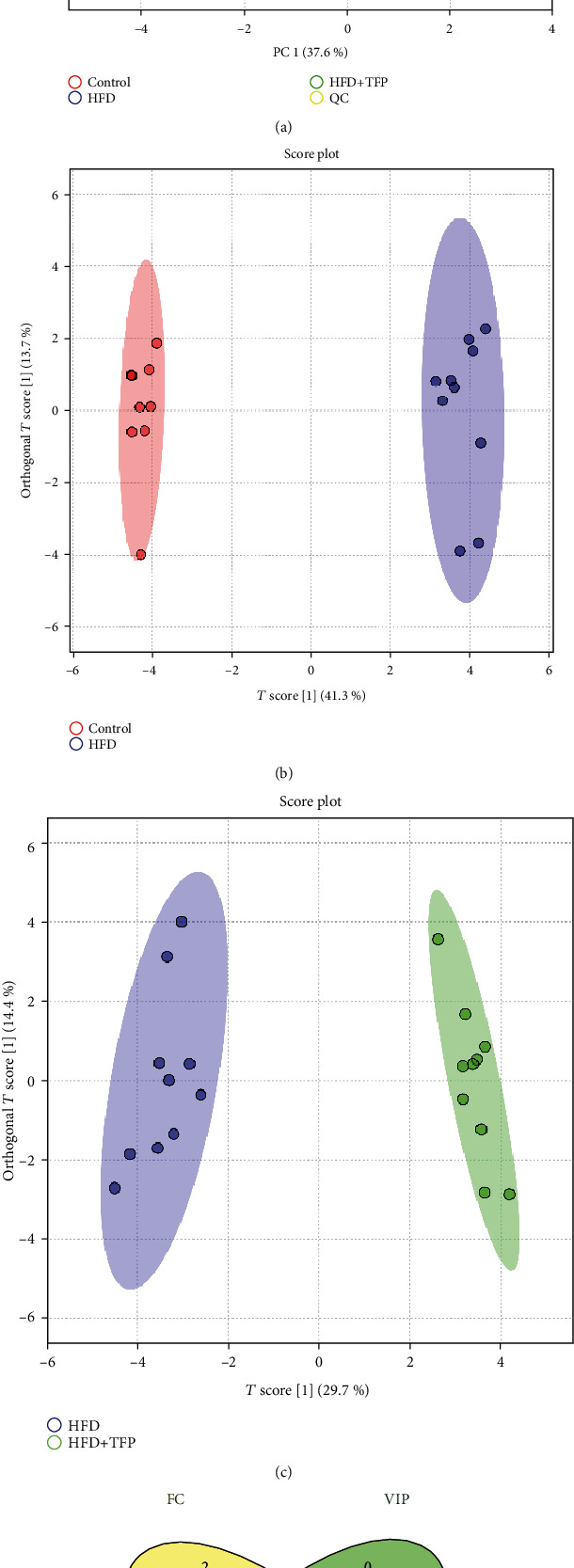
(a) PCA score plot of four treatments, exhibiting a clear distinction among four groups. (b, c) OPLS-DA score plots of the control group versus HFD group and HFD group versus HFD+TFP group. Their model parameters are *R*^2^*Y* = 0.893 and *Q*^2^*Y* = 0.866 and *R*^2^*Y* = 0.859 and *Q*^2^*Y* = 0.787, respectively. (d) Venn analysis result based on FC, VIP, *P* value, and ROC analysis.

The total ion chromatograms of serum samples from the control, HFD, and HFD+TFP groups are displayed in Figure 4. After data processing, multivariate statistical analysis was performed on the website MetaboAnalyst 5.0 (http://www.metaboanalyst.ca/). QC samples were used to check the robustness associated with sample processing and experimental conditions and guarantee the accuracy of data obtained.  Table 1 illustrates that the analytical process was reliable, as the RSD values of all peaks in QC samples were lower than 30%. Moreover, an unsupervised PCA model was constructed to assess the stability and reliability of the analytical method and the classification trends among treatments. As illustrated in Figure 5(a), QC samples were clustered (all spots near the central point), indicating that the data obtained were reliable. Meanwhile, the HFD group samples were quite different from the control group samples, demonstrating that the HFD group presented a distinct metabolic profile compared with the control group. Besides, the grouping trend of samples was found to exhibit the reversal effect of TFP treatment, which was consistent with the result of the biochemical examination.

On the contrary, 42 metabolites were identified, including organic acids, long-chain fatty acids, acetone bodies, and carbohydrates. As shown in Table 1, the *P* value and FC were applied to exhibit the intergroup differences and the trend of metabolites between groups. The results showed that amino acids (such as norvaline, serine, L-alanine, and threonine), carbohydrates (such as D-(+)-galactose and tagatose), trans-9-octadecenoic acid, cholesterol, and urea had the maximum concentration variations (significantly upregulated in the HFD group compared with the control group but downregulated in the HFD+TFP group compared with the HFD group). It further manifested that the alteration in the metabolic mechanism of HFD-induced hyperlipidemia rats was probably related to the metabolic mechanism of amino acids, carbohydrates, trans-9-octadecenoic acid, cholesterol, and urea. Subsequently, the pathway analysis based on potential biomarkers explained the changes in metabolic mechanisms responsible for these components.

#### 3.6.2. Pattern Recognition Analysis of Metabolic Profiling among Different Treatments

The supervised OPLS-DA model was constructed to exhibit a maximum separation among groups and screen differential metabolites to find differential metabolites of discrimination of treatments and unravel the influence of TFP on the metabolic profiles of rats in *vivo*. As shown in Figures 5(b) and 5(c), the OPLS-DA model was constructed between the control and HFD groups using the model parameters (*R*^2^*Y* = 0.893, *Q*^2^*Y* = 0.866), while the OPLS-DA model presented between the HFD and HFD+TFP groups using the model parameters (*R*^2^*Y* = 0.859, *Q*^2^*Y* = 0.787). *R*^2^*Y* illustrated the explanatory ability of the model, indicating that the OPLS-DA model could elucidate the intergroup separation. Moreover, the high predictive power evidenced by the parameters with *Q*^2^*Y* > 0.5 provided evidence for the discrimination between groups. Likewise, a tenfold cross-validation and permutation test (*n* = 100 times) were performed to examine the overfitting of models; the result showed no overfitting (*P* < 0.05). These results indicated that the models were reliable.

#### 3.6.3. Identification of Potential Biomarkers on Hyperlipidemia

The metabolites with VIP > 1.0 and *P* < 0.05 were identified tentatively as the potential biomarkers (Table 1). The result showed that 17 metabolites were selected as the potential biomarkers from the OPLS-DA model constructed based on the control group versus the HFD group, while 11 metabolites were selected based on the HFD group versus the HFD+TFP group. Subsequently, the ROC curve associated with all metabolites were drawn, and the metabolites with the AUC‐ROC > 0.90 were selected as potential biomarkers [24]. The ROC curve also confirmed the suitability of metabolites as potential biomarkers based on the differential metabolites screened from the aforementioned OPLS-DA models with VIP > 1.0 and *P* value < 0.05. The method using VIP and *P* values had an excellent accuracy in terms of biomarker screening since the AUC-ROC of all differential metabolites exceeded 0.80. Seven metabolites were screened out as intersectional differential metabolites based on the VIP, *P*, and FC values (FC > 1.5). They were norvaline, L-alanine, urea, cholesterol, trans-9-octadecenoic acid, threonine, and serine, exhibited through constructing the Venn diagram (Figure 5(d)).

**Figure 6:**
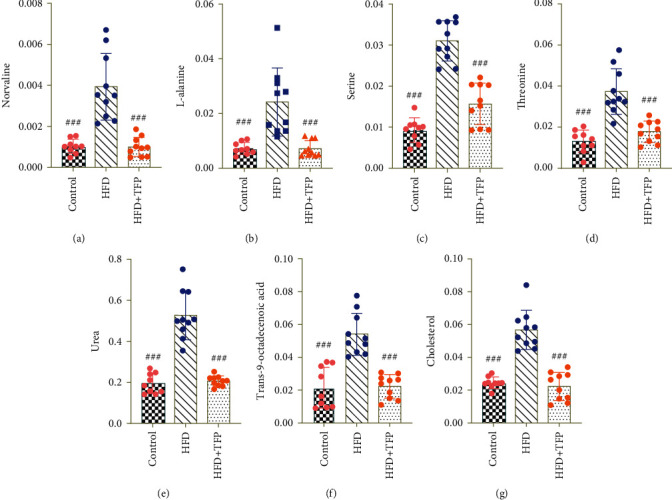
Variations in the trends of the metabolites that are biomarkers of differential treatments (a–g). They showed the variations in the trends of norvaline, L-alanine, urea, cholesterol, trans-9-octadecenoic acid, threonine, and serine, respectively. ^###^*P* < 0.001, ^##^*P* < 0.01, and ^#^*P* < 0.05 compared with the HFD group.

The correlation analysis of the seven potential biomarkers was performed to unravel the interconnection of these biomarkers. The result showed a positive correlation between L-alanine and norvaline, urea and cholesterol, threonine and trans-9-octadecenoic acid, urea and norvaline, and norvaline and threonine (correlation coefficient = 0.820, 0.809, 0.786, 0.758, and 0.745, respectively). Specifically, as shown in Figure 6, all potential biomarkers displayed dramatic changes before and after TFP administration. The seven metabolites were significantly upregulated by the HFD compared with those in the control group and markedly downregulated by TFP compared with those in the HFD group.

#### 3.6.4. Metabolic Pathway Analysis and Enrichment Analysis

**Figure 7:**
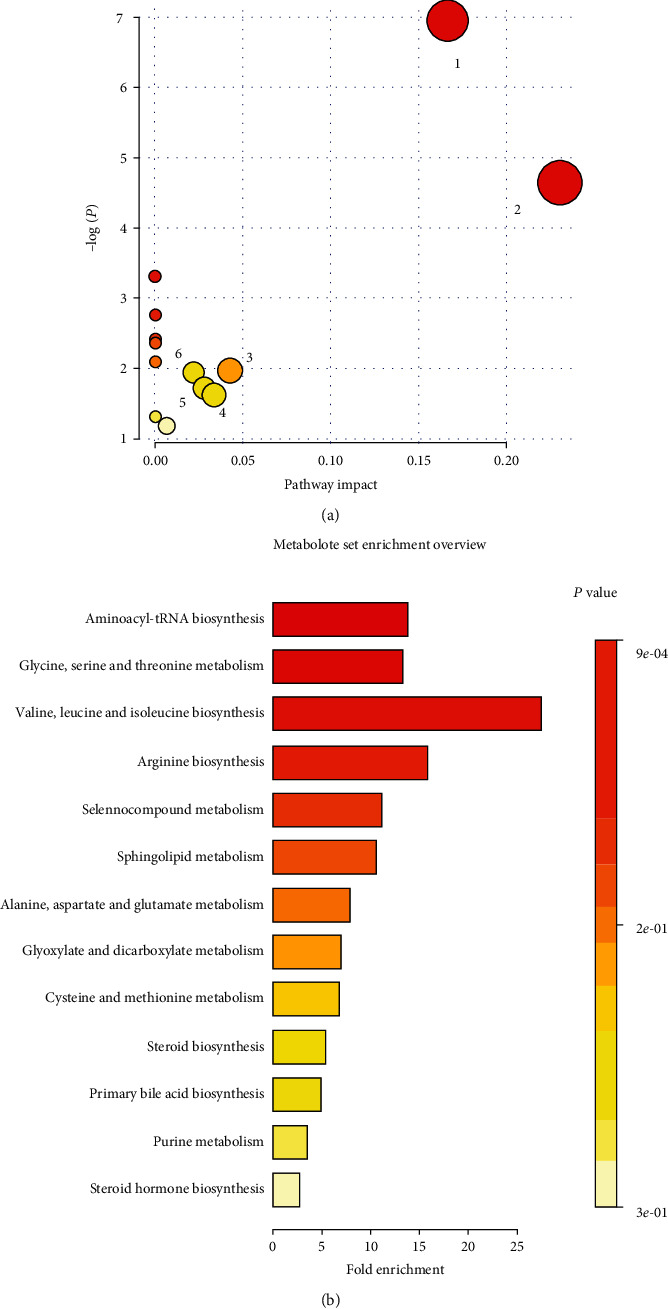
(a) Pathway analysis of 7 potential biomarkers: (1) aminoacyl-tRNA biosynthesis and (2) glycine, serine, and threonine metabolism. (b) Enrichment analysis of 7 potential biomarkers; a higher position and deeper colour demonstrated a strong correlation between the enriched metabolic pathways.

The threshold of the impact value of the pathway analysis using MetaboAnalyst 5.0 was set to 0.10 for finding the most relevant potential metabolic pathway related to the liver-protective effects of TFP. The seven potential biomarkers associated with group separation were imported into the online website, which combined the topology with a powerful pathway and enrichment analysis. The result of pathway analysis showed that the aminoacyl-tRNA biosynthesis and glycine, serine, and threonine metabolism were the main impacts (Figure 7), although glyoxylate and dicarboxylate metabolism, primary bile acid biosynthesis, steroid biosynthesis, and cysteine and methionine metabolism also had some contribution (Table 2). The metabolic pathways with *P* < 0.05 (aminoacyl-tRNA biosynthesis and glycine, serine, and threonine metabolism) were identified as differential metabolic pathways by enrichment analysis. The aforementioned results indicated that the most related metabolic pathways were glycine, serine, and threonine metabolism and aminoacyl-tRNA biosynthesis. They were observed in both the pathway analysis and the pathway enrichment analysis. Therefore, the two pathways were confirmed as important links to HFD-induced hyperlipidemia and the lipid-lowering effect of TFP.

#### 3.6.5. Signaling Networks

**Figure 8:**
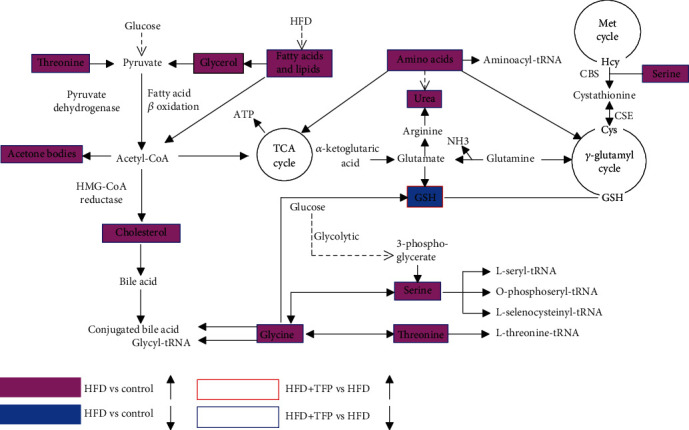
Schematic diagram of the related metabolic pathway. The pink and light blue boxes indicate metabolites significantly higher and lower in the HFD group than in the control group, respectively. The boxes bordered in red and blue represent metabolites significantly higher and lower in the HFD+TFP group than in the HFD group, respectively.

Potential biomarkers and related biological pathways were imported into Kyoto Encyclopedia of Genes and Genomes (KEGG) (http://www.kegg.jp/) to find interactions, revealing the relationships among these signaling pathways. The networks were primarily related to glycine, serine, and threonine metabolism and aminoacyl-tRNA biosynthesis (Figure 8), suggesting that the ameliorating effect of TFP on rats with hyperlipidemia was significantly related to the two pathways.

## 4. Discussion

The present study showed that an HFD produced dramatic metabolic alterations in hyperlipidemia, but TFP ameliorated the change considerably. The changes in the metabolic profiles were consistent with the results of biochemical indicator measurements and histological observation. TFP significantly reduced the HFD-induced abnormal lipid levels, apart from the lowering effects on the serum levels of ALT and AST and the amelioration effects on the oxidative stress indicators and inflammatory factors. The amelioration of histological features by TFP implied that the intake of TFP might be beneficial to prevent and improve HFD-induced lipid disorder. Furthermore, the result of metabolomic analysis suggested that TFP prevented and improved the HFD-induced lipid disorder primarily through regulating the seven metabolites and the main processes: glycine, serine, and threonine metabolism and aminoacyl-tRNA biosynthesis.

Studies showed that the increase in liver enzyme activities in serum was attributed to the leakage of cellular enzymes into the plasma [33]. The elevation of serum ALT (mainly distributed in the cytoplasm of hepatocytes) and AST (mainly distributed in the cytoplasm and mitochondria of hepatocytes) levels reflects the damage to the hepatocyte membrane, hepatocytes, and organelles [30]. In the present study, the suppression of ALT and AST activities was observed in the HFD+TFP group, indicating the potential effects of TFP in preserving the structural integrity of hepatic cells by increasing the stabilization of the plasma membrane [34].

The pathogenic progress of hyperlipidemia could be accelerated by oxidative stress usually caused by the excessive production of reactive oxygen species (ROS) [35, 36]. When cellular ROS overwhelmed antioxidant capacity, damage to cellular macromolecules such as lipids may ensue [37, 38]. SOD and GSH levels were regarded as biomarkers reflecting the production of free radicals and the primary defense system against ROS. They could defend against ROS formation and convert active oxygen molecules into nontoxic compounds, reducing oxidative stress [39]. In the present study, the alterations in SOD, GSH, and MDA levels in the HFD group were significantly ameliorated by TFP, indicating that TFP participated in regulating the antioxidant enzyme system. The hepatoprotective effect of TFP was also confirmed in histological investigations. This evidence suggested that the hepatoprotective effects of TFP might be linked to their antioxidant and prooxidant properties [40], and polysaccharides exerted their positive functions by scavenging free radicals [41]. It was inferred that the significant antioxidant activity of TFP *in vivo* might be through the regulation of antioxidant enzymes, which could scavenge the overproduced oxygen-free radicals.

GSH is the primary antioxidant. Its synthesis is related to the *γ*-glutamyl cycle, which is responsible for the intake of amino acids. In the cycle, glycine has an important role. Glycine is widely accepted as an antioxidant and cytoprotective substance [42]. It is a major source of methyl groups from the one-carbon pool required for the biosynthesis of GSH in aerobic cells [43]. Studies have shown that glycine has the potential to act as a hepatospecific antioxidant to reduce oxidant and cytokine production by Kupffer cells and promote hepatic fatty acid oxidation [44]. Moreover, threonine and serine can also be transformed into glycine in the synthesis of GSH under the action of related enzymes [45] (Figure 8). When the synthesis of GSH from glycine is blocked, severe oxidative stress occurs due to the lack of GSH. The rats in the HFD group had a high glycine level and a low GSH level, as well as severe oxidative stress injury compared with the control group. This finding suggested that the synthesis of GSH from glycine was blocked, further resulting in glycine accumulation and deficiency of GSH [46]. The significant ameliorating effect of TFP on GSH and serum glycine levels in the HFD group indicated that TFP could protect liver cells from oxidative stress by promoting the synthesis of GSH from glycine. Studies also suggested that glycine cytoprotection for a variety of cells was activated through the glycine receptor (GlyR) and glycine transporter-1 (GlyT1) [47]. Therefore, the hepatoprotective effect of TFP might be associated with the activation of the GlyTs and GlyR for promoting the intake of glycine. Besides, the decrease in the number of amino acids in the HFD+TFP group might also be related to the promoting effect of TFP on the formation of aminoacyl-tRNA.

Further, the synthesis of GSH is also related to the serine level *in vivo* (Figure 8). Except for the exogenous supply, serine is biosynthesized *de novo* from a glycolytic intermediate 3-phosphoglycerate [43]. The transsulfuration pathway is an endogenous pathway to use homocysteine (Hcy) for the production of GSH. The transsulfuration pathway of serine is of importance in the maintenance of redox homeostasis [48]. Previous studies showed that the hyperlipidemia led to a significant decrease in cystathionine *β*-synthase (CBS) and cystathionine *γ*-lyase (CSE) activities in the liver and blocked the Hcy conversion to Cys, resulting in not only the upstream accumulation of Hcy but also downstream deficiency of Cys and subsequent reduction of antioxidant GSH synthesis [49]. Besides, serine also decreased the serum levels of ALT, AST, LDL-C, TG, hepatic MDA, and ROS, apart from the increase in SOD level, oxidation of fatty acids, glucose tolerance, and insulin sensitivity in HFD-induced mice [50]. Serine could condense with Hcy to synthesize Cys and thus alleviate oxidative stress via supporting GSH synthesis [51], and the decrease in CBS and CSE activities in rats with HFD-induced hyperlipidemia caused the upstream accumulation of Hcy and the lack of downstream GSH [49]. In this study, the high serine and low GSH levels were probably due to the HFD-induced low activity of CBS and CSE, resulting in the accumulation of serine and the lack of GSH. A part of the potent antioxidant effect of TFP was a result of its activatory effect on the transsulfuration pathway, accounting for the ameliorating effect of TFP on the GSH level in the liver. The activation of the transsulfuration pathway of serine by TFP via changing the activities of the target enzymes (CBS and CSE) needs further exploration.

As for the limitation of the study, (1) the focus tissue samples are not available now so that the metabolic test for lesion tissue was unavailable. However, the blood samples for metabolism were convincing theoretically since they may contain more biomarkers compared with organs and tissues. (2) The effect of TFP on normal rats was not investigated in this experiment. However, the natural polysaccharides are generally nontoxic and have no oxidation-promoting effect according to many studies. (3) As to the determination of indicators of oxidative stress, the experiment has been done for a long time, and the total protein content of the sample was not measured because we did not take into consideration the fact that the results of oxidation determination should be standardized to mg of total protein. It is also one of the limitations of this study.

## 5. Conclusion

In conclusion, this study demonstrated that TFP intervention reduced body weight, oxidative stress, and inflammatory conditions, improved liver injury symptoms, and reversed HFD-induced dyslipidemia. The metabolomic results indicated that the mechanism underlying the lipid-lowering effect of TFP was associated with the regulation of the antioxidant enzyme system and the transformation of amino acids into aminoacyl-tRNA. Therefore, the results indicated that TFP might be developed as a natural hypolipidemic supplement for preventing hyperlipidemia.

## Figures and Tables

**Table 1:** Identified metabolites related to hyperlipidemia based on GC-MS.

	Metabolites	Ret. time (min)	RSD of QC (*n* = 6)	HFD vs. control			HFD+TFP vs. HFD		
No.				*P* value	FC	AUC	*P* value	FC	AUC
1	Tagatose	19.652	9.153%	≤0.001	2.862	0.98	<0.05	0.631	0.73
2	Fructose	19.794	4.802%	<0.005	1.809	0.87	≤0.001	0.526	0.91
3	D-Mannose	19.876	5.981%	>0.05	1.199	0.70	>0.05	0.913	0.60
4	Glucose	20.020	2.706%	<0.01	1.270	0.83	<0.05	0.808	0.82
5	Allose	20.305	1.660%	≤0.001	2.660	1.00	>0.05	0.853	0.75
6	D-(-)-Ribose	16.907	7.565%	<0.05	1.324	0.80	<0.05	0.753	0.81
7	D-(+)-Galactose	21.723	11.369%	≤0.001	2.365	0.94	<0.01	0.529	0.89
8	Propanoic acid	7.098	3.604%	≤0.001	1.481	0.90	<0.01	0.749	0.83
9	Acetic acid	7.363	4.816%	>0.05	1.227	0.66	>0.05	0.766	0.73
10	Butanoic acid	8.159	5.728%	>0.05	0.926	0.43	<0.05	0.784	0.82
11	DL-*β*-Hydroxybutyric acid	8.718	4.097%	<0.05	1.262	0.82	<0.01	0.697	0.90
12	Succinic acid	11.247	7.611%	>0.05	1.388	0.70	>0.05	0.897	0.50
13	Glyceric acid	11.463	5.898%	<0.05	1.470	0.77	>0.05	0.872	0.57
14	2,3-Dihydroxybutanoic acid	11.670	6.510%	>0.05	1.235	0.68	>0.05	0.850	0.66
15	Aminomalonic acid	13.750	15.590%	<0.01	1.533	0.89	<0.01	0.655	0.86
16	Trans-9-octadecenoic acid	24.160	7.442%	≤0.001	2.515	1.00	≤0.001	0.412	1.00
17	Stearic acid (17 : 0)	24.470	6.800%	≤0.001	1.798	1.00	<0.01	0.723	0.90
18	Hexadecanoic acid	22.083	4.731%	≤0.001	1.709	1.00	<0.05	0.868	0.75
19	Threonic acid	15.133	6.778%	≤0.001	2.339	0.99	<0.01	0.559	0.83
20	Cis-5,8,11-eicosatrienoic acid	25.886	21.165%	<0.01	1.807	0.81	<0.05	0.647	0.76
21	L-Alanine	7.790	8.732%	≤0.001	3.361	1.00	≤0.001	0.294	0.99
22	Norvaline	9.623	24.629%	≤0.001	3.821	1.00	≤0.001	0.254	1.00
23	Serine	10.321	7.818%	≤0.001	3.338	1.00	≤0.001	0.506	1.00
24	Threonine	10.923	10.560%	≤0.001	2.850	1.00	≤0.001	0.475	0.97
25	Glycine	11.117	7.384%	≤0.001	1.674	0.93	<0.05	0.767	0.79
26	L-5-Oxoproline	14.613	3.749%	<0.05	1.492	0.81	<0.01	0.646	0.80
27	Undecane	8.882	7.040%	>0.05	1.190	0.68	>0.05	0.836	0.60
28	Dodecane	9.474	7.120%	<0.05	1.673	0.84	<0.05	0.652	0.79
29	Pentacosane	10.703	5.265%	<0.01	1.603	0.89	>0.05	0.800	0.68
30	Hexadecane	17.410	6.431%	<0.01	1.552	0.88	>0.05	0.910	0.57
31	Eicosane	23.076	5.808%	≤0.001	2.746	1.00	>0.05	0.819	0.65
32	Undodecane	7.836	6.233%	<0.05	1.553	0.90	<0.05	0.709	0.73
33	Inositol	22.550	7.521%	>0.05	1.072	0.58	>0.05	0.890	0.59
34	Cholesterol	26.403	8.750%	≤0.001	2.309	1.00	≤0.001	0.393	1.00
35	2-Hydroxypyridine	6.743	16.887%	>0.05	1.315	0.80	>0.05	0.754	0.73
36	5,6-Dihydrouracil	8.406	1.620%	<0.05	1.384	0.76	<0.01	0.663	0.86
37	Urea	10.126	7.550%	≤0.001	2.709	1.00	≤0.001	0.392	1.00
38	Glycerol	10.548	5.632%	>0.05	1.176	0.68	>0.05	0.891	0.63
39	Phosphate	10.584	4.855%	≤0.001	1.687	0.98	<0.01	0.732	0.94
40	3-Aminopropionitrile	11.850	6.541%	>0.05	1.370	0.70	>0.05	0.684	0.72
41	1,5-Anhydrosorbitol	19.450	5.124%	≤0.001	1.746	0.91	<0.05	0.760	0.78
42	Tetracosane	25.523	12.435%	>0.05	1.037	0.50	>0.05	0.998	0.45

**Table 2:** Result from ingenuity pathway analysis with MetaboAnalyst 5.0.

Pathway name	Match status	Raw *P*	−Log(*P*)	Impact
Aminoacyl-tRNA biosynthesis	3/48	9.6749*E* − 04	6.9408	0.16667
Glycine, serine, and threonine metabolism	2/34	0.0096437	4.6415	0.23069
Valine, leucine, and isoleucine biosynthesis	1/8	0.036597	3.3078	0.0
Arginine biosynthesis	1/14	0.063286	2.7601	0.0
Selenocompound metabolism	1/20	0.089339	2.4153	0.0
Sphingolipid metabolism	1/21	0.09362	2.3685	0.0
Alanine, aspartate, and glutamate metabolism	1/28	0.12311	2.0947	0.0
Glyoxylate and dicarboxylate metabolism	1/32	0.13959	1.9691	0.04233
Cysteine and methionine metabolism	1/33	0.14366	1.9403	0.02184
Biosynthesis of unsaturated fatty acids	1/36	0.1558	1.8592	0.0
Steroid biosynthesis	1/42	0.17962	1.7169	0.0282
Primary bile acid biosynthesis	1/46	0.19519	1.6338	0.03324
Purine metabolism	1/66	0.26926	1.3121	0.0
Steroid hormone biosynthesis	1/77	0.30745	1.1794	0.00662

The matching status means the ratio of the number of compounds in the pathway to the matched number from the user uploaded data; the raw *P* is the original *P* value calculated from the enrichment analysis; the impact is the pathway impact value calculated from the pathway topology analysis.

## Data Availability

The raw data used to support the findings of this study are available from the corresponding author upon request.

## Abbreviations

GC-MS: Gas chromatography-mass spectrometer
TFP: Polysaccharide from *Turpiniae folium*
TC: Total cholesterol
TG: Triglyceride
LDL-c: Low-density lipoprotein cholesterol
HDL-c: High-density lipoprotein-cholesterol
ELISA: Enzyme-linked immunosorbent assay
TNF-*α*: Tumor necrosis factor *α*
BSTFA: *N*,*O*-Bis(trimethylsilyl) trifluoroacetamide
SPF: Specific pathogen free
SD: Sprague-Dawley
HFD: High-fat diet
HSI: Hepatosomatic index
ALT: Alanine aminotransferase
AST: Glutamic oxaloacetic transaminase
ALP: Alkaline phosphatase
MDA: Malondialdehyde
GSH: Glutathione
SOD: Superoxide dismutase
CV: Coefficient of variation
QCs: Quality control samples
MS-DAIL: Mass Spectrometry-Data Independent Analysis
RSD: Relative standard deviation
PCA: Principal component analysis
OPLS-DA: Orthogonal projection to latent structure discriminant analysis
FC: Fold change
AUC-ROC: Area under the receiver operating characteristic
VIP: Projection importance of characteristic variable
Cys: Cysteine
KEGG: Kyoto Encyclopedia of Genes and Genomes
ROS: Reactive oxygen species
GlyR: Glycine receptor
GlyT1: Glycine transporter-1
Hcy: Homocysteine
CBS: Cystathionine *β*-synthase
CSE: Cystathionine *γ*-lyase.
